# Hybrid immunity strategies: heterologous vaccination combined with natural SARS-CoV-2 infection

**DOI:** 10.3389/fmed.2026.1828887

**Published:** 2026-04-21

**Authors:** Yuan Huang, Xiaolu Zhang, Ruixia Miao, Jing Wang, Xuan Xi, Heng Pan, Yan Lu, Dehong Li

**Affiliations:** 1 Department of Public Health, Gansu University of Chinese Medicine, Lanzhou, China; 2 Department of Clinical Laboratory, Gansu Provincial Hospital, Lanzhou, China; 3 Department of Blood Transfusion, Gansu Provincial Hospital, Lanzhou, China

**Keywords:** antigenic distance, heterologous strategies, hybrid immunity, immune memory, SARS-CoV-2 variants

## Abstract

The continuous evolution of SARS-CoV-2 poses a significant challenge to existing immune barriers, highlighting the limitations of single-modality immunization. Hybrid immunity, shaped by the combination of natural infection and vaccination, induces more potent, broad-spectrum, and durable protective immunity. This review proposes that heterologous vaccination – the sequential use of vaccines based on different technological platforms – can serve as an active public health strategy to simulate and optimize hybrid immunity. We systematically elaborate on the synergistic advantages of hybrid immunity at both the humoral and cellular levels, citing evidence from multiple clinical trials that for both convalescent individuals and infection-naïve populations, heterologous vaccination regimens outperform homologous regimens in enhancing the breadth of neutralizing antibodies, strengthening cross-protection, and establishing robust immune memory. By mimicking the antigenic distance effect, heterologous vaccination safely replicates the immunological benefits of natural infection without the associated risks, positioning it as a key strategic tool to counter persistent viral evolution and build a resilient population-wide immune barrier.

## 1 Introduction

The successive emergence of SARS-CoV-2 Variants of Concern (VOCs) has not only increased viral transmissibility but also led to a significant reduction in neutralizing antibody titers induced by ancestral strain-based vaccines and prior infections, resulting in substantial immune escape. Consequently, even individuals with pre-existing immunity remain susceptible to breakthrough infections. The rapid evolution of the virus has exposed the inherent limitations of single-modality immune stimulation: both homologous vaccination and single natural infection exhibit waning efficacy over time in preventing infection and confer a relatively narrow protective spectrum (1, 2). To address this challenge, second-generation vaccines (such as bivalent vaccines targeting the ancestral strain and Omicron BA.4/5, and subsequent monovalent XBB vaccines) have been developed, aiming to enhance neutralization breadth against emerging variants. However, despite demonstrating improved immunogenicity, the ultimate real-world protective efficacy of these second-generation vaccines remains highly dependent on an individual’s complex prior immune history (3).

**Figure 1:**
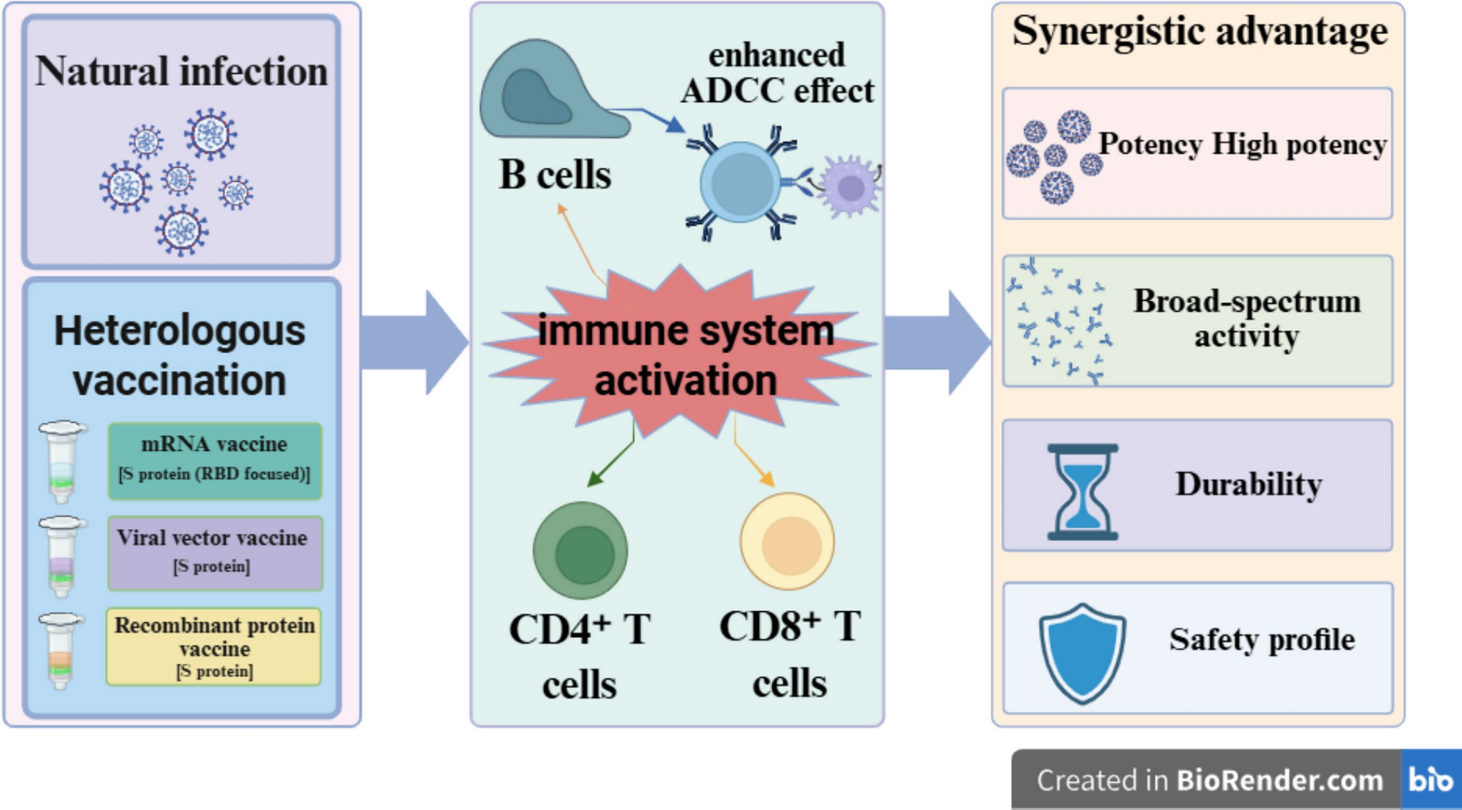
Schematic illustration of the synergistic mechanism by which heterologous vaccination simulates and optimizes hybrid immunity. This figure illustrates the synergistic mechanism by which heterologous vaccination enhances and optimizes hybrid immunity. The upper left section depicts natural infection, which provides multi-antigenic stimulation involving the full virus, including the nucleocapsid (N) protein. The lower left section represents heterologous vaccination—using platforms such as mRNA, viral vector, and recombinant protein vaccines—that delivers spike (S) protein antigens, thereby introducing antigenic distance. These two pathways converge to co-activate the central immune system, including B cells, CD4^+^ T cells, and CD8^+^ T cells. This leads to the induction of highly cross-reactive neutralizing antibodies and antibody-dependent cellular cytotoxicity (ADCC), while also establishing robust memory T cell responses. Ultimately, this synergistic interplay results in the core advantages of hybrid immunity illustrated on the right: high potency, broad-spectrum activity, durability, and a favorable safety profile. This image was created using BioRender (http://biorender.com/; accessed on April 14, 2026), and is reproduced with permission from BioRender.

In this review, hybrid immunity – the immune state induced by the combination of natural infection and vaccination – has become a research focus due to its ability to elicit stronger and more durable immune protection (4). To translate this passively observed immunological advantage into a proactive public health strategy, this review proposes the adoption of an active immunization regimen that integrates the broad immune memory established by natural infection with heterologous boosting using vaccines from distinct technological platforms. This strategy aims to construct a reinforced immune profile with both breadth and precision, with the potential to overcome the limitations of single-modality immunization and provide a more resilient defense against ongoing viral evolution. Against this background, this review systematically argues for the significant potential of heterologous vaccination as a proactive public health strategy in simulating and optimizing hybrid immunity in response to the persistent evolution of SARS-CoV-2. A growing body of evidence indicates that this strategy is significantly superior to traditional homologous regimens in inducing broad-spectrum, potent, and durable humoral and cellular immune responses, a conclusion validated in both convalescent and infection-naïve populations (5, 6). As Parker et al. have noted in a seminal commentary, heterologous vaccination, initially adopted out of necessity, may represent a strategic direction for optimizing immune responses and broadening protection in the context of variant emergence and vaccine supply variability (7). Figure 1 provides a schematic representation of the core mechanism by which heterologous vaccination simulates and optimizes hybrid immunity, serving as an intuitive foundation for the more specific arguments presented in the following sections.

## 2 Hybrid immunity: synergistic effect of antigen exposure drives superior immune protection

A comprehensive understanding of the synergistic effects of hybrid immunity requires examination within the evolving landscape of population immunity (8). Previously, protective immunity was primarily achieved through two independent, single-modality approaches: natural infection alone or homologous vaccination (9). Natural infection, as the primordial immunization route, presents the immune system with a wide array of viral antigens, including the nucleocapsid (N) protein, eliciting a diverse T-cell response; however, the strength and durability of this immunity are highly heterogeneous and come with risks of acute severe disease and long-term sequelae (10). In contrast, homologous vaccination targeting the spike (S) protein offers a safer, more controlled alternative, inducing standardized, high-titer neutralizing antibody responses. However, it may generate overly focused immune responses, limiting breadth and increasing susceptibility to immune imprinting effects against evolving variants (11). Heterologous vaccination strategies, initially adopted out of necessity, have since been recognized as a preferred option for boosting. By leveraging the antigenic distance between different vaccine platforms to actively broaden the immune response, these strategies represent an effective means of artificially designing and optimizing immune status. Notably, recent research employing quantitative biological models, such as antigenic fields, further supports the antigenic distance’ concept, indicating that rationally designed heterologous boosting based on this principle can facilitate the induction of broader immune protection (12). A substantial body of evidence indicates that hybrid immunity demonstrates the potential to overcome the inherent limitations of single-modality immunization approaches, positioning it as a promising new paradigm (8). It not only produces additive effects but, through the synergistic action of different antigen exposures, induces immune protection that is more powerful, broad-spectrum, and durable than that elicited by single exposures (9, 13). The superiority of hybrid immunity stems from the synergistic and enhancing effects generated within the immune system through exposure to antigens in different forms, which is specifically reflected in two dimensions: the breadth of antigen recognition and the intensity and durability of the immune response (14).

### 2.1 Breadth of antigen recognition: complementarity of whole virus and targeted protein

From the perspective of antigen recognition breadth, hybrid immunity arises from dual exposure: natural infection targeting variant-specific antigens and vaccination against ancestral or updated vaccine strains. On one hand, this enables cross-reactive memory B cells induced by natural infection to broaden their recognition range against variants such as Beta, Delta, and Omicron upon vaccine activation. On the other hand, it directs the induced CD8^+^ memory T cells to target conserved epitopes of the S protein, while CD4^+^T cells recognize non-S conserved antigens such as the N protein, thereby effectively countering immune evasion by variants (15). This mechanism is supported by the study of Buckner et al., which confirmed that hybrid immunity elicits broadly cross-reactive B cells and antibodies in terms of antigen recognition breadth, significantly enhancing coverage against multiple variants (16). Furthermore, this enhancement in recognition breadth stems from the complementary effects of natural infection and vaccination in antigen exposure: Natural infection provides multi-epitope antigens derived from the whole virus, including the N protein, activating broad CD4 + and CD8 + T-cell responses (17). Vaccination, conversely, focuses on the S protein, particularly the RBD domain, reinforcing humoral immunity against key infection targets through high-purity, high-dose antigen delivery (18). Their combination creates an immune recognition landscape that balances breadth and precision, and this complementarity is particularly evident in the antibody response. A study by Grant et al. showed that hybrid immunity synergistically elicits combined antibody responses against the S1 and S2 domains of the spike protein, significantly enhancing antibody-dependent cellular cytotoxicity (ADCC) and conferring broader protection against SARS-CoV-2 variants (19).

### 2.2 Immune response potency and durability: optimizing B and T cell memory

Hybrid immunity significantly enhances the magnitude of the immune response. Compared with unvaccinated convalescent individuals, convalescent vaccinees exhibit a 50-fold higher neutralizing antibody level, accompanied by an increased frequency and cytotoxicity of S-specific CD8^+^T cells and the induction of unique anti-inflammatory protective bifunctional CD4^+^T cells. Furthermore, hybrid immunity substantially prolongs the duration of protection. This is reflected in the stable maintenance of neutralizing antibodies for over 6 months with slow decay, nasal mucosal immune protection lasting up to 10 months, memory CD8^+^and CD4^+^T cell half-lives exceeding 190 days, and memory B cells that can be rapidly reactivated by vaccination even two years post-infection (15). This provides superior intensity and durability benefits from the optimization of the immune response through sequential exposure to different forms of antigens. Sequential exposure to antigens in different forms profoundly optimizes the quality of the immune response. These antigens consist of variant antigens derived from natural infection and wild-type or updated vaccine strain antigens provided by vaccination. In humoral immunity, it not only stimulates higher levels of neutralizing antibodies but also drives B-cell affinity maturation and epitope spreading, generating a more diverse repertoire of memory B cells with higher affinity and broader neutralizing capacity (20). In cellular immunity, hybrid immunity establishes more robust T-cell memory targeting conserved viral regions, which can effectively prevent severe disease even against new variants capable of antibody escape (21). Providing further support for this perspective, data from the study by Ford et al. demonstrate that in previously infected individuals, subsequent mRNA vaccination supports the expansion and diversification of the S-specific CD8^+^T cell repertoire. The superior protective efficacy may stem from a stepwise enhancement of response intensity, continuous replenishment of new clones, and the persistence of vaccine-expanded clones (22). These findings collectively corroborate the conclusions of the study by Rodda et al., namely that hybrid immunity, shaped by both natural infection and vaccination, can induce more robust and durable immune memory than any single immunization approach, and that the unique functional imprint on T cells conferred by natural infection is difficult to fully replicate through homologous vaccination alone (23). In summary, as emphasized by Buckner et al., optimizing the timing of antigen exposure enables the induction of more robust and durable immune memory in terms of response intensity and durability (16). This optimized memory repertoire responds more rapidly and decays more slowly, mechanistically explaining why hybrid immunity provides more durable and powerful protection.

## 3 Heterologous vaccination: from theory to clinical application

Relying on unpredictable and risky natural infection to achieve this superior immune state of hybrid immunity is not a feasible public health strategy. Meanwhile, traditional homologous boosting strategies (using the same vaccine platform) have shown limitations: although they effectively reactivating existing immune memory, such approaches tend to primarily reinforce responses against the ancestral antigen. This phenomenon known as immune imprinting, can limit the breadth of the immune response against new variants (20). Therefore, to actively and safely replicate the benefits of hybrid immunity, we propose employing heterologous vaccination – the sequential administration of vaccines based on different technological platforms – as a core strategic tool. This strategy aims to simulate the broad immune activation akin to natural infection by introducing antigenic distance generated by different vaccine platforms, while avoiding its associated risks. By combining a broad immunological foundation (established via prior infection or heterologous priming) with multi-platform booster vaccination, a theoretical framework emerges for constructing an immune profile that balances breadth and potency. Current evidence supports the potential of this approach in addressing persistent viral evolution and could contribute to a more resilient population-wide immune barrier (3, 5, 6, 24).

This theoretical framework is supported by real-world evidence. A 2022 review by Parker et al. (Lancet Infect Dis) encompassing 48 studies found that heterologous schedules, particularly vectored-mRNA combinations, generally elicit immune responses comparable or superior to homologous regimens, with effectiveness of approximately 90% or higher against severe disease. Although short-term reactogenicity may be increased, the overall safety profile appears acceptable, offering supportive evidence for heterologous vaccination as a strategy to optimize hybrid immunity (25). Heterologous vaccination strategies have demonstrated superior application value in individuals with different immune backgrounds, specifically building and optimizing immune protection in convalescent individuals, infection-naive populations, and those experiencing breakthrough infections.

### 3.1 Convalescent individuals: synergistic and enhanced effects of heterologous booster vaccination

For individuals with prior infection, heterologous boosting can significantly leverage the broad immune foundation established by natural infection, achieving synergistic enhancement that builds upon prior immunity. Awadalla et al. (5) conducted a one-year follow-up study of 484 subjects (including 214 with prior infection), finding that hybrid immunity induced by heterologous boosting (mRNA + viral vector vaccines) combined with natural infection was superior to homologous boosting in both humoral immunity (anti-S IgG levels, neutralization inhibition against Omicron B.1.1.529/BA.2) and cellular immunity (CD8^+^ T-cell IFN-*γ* response). Two doses of heterologous vaccination combined with infection achieved protection levels comparable to three doses of homologous vaccination, suggesting advantages for this strategy in enhancing immune durability, extending protection against variants, and reducing the number of vaccine doses required (5). Research by Elliott et al. further revealed that in convalescent individuals, priming with saRNA followed by an mRNA booster not only induced higher neutralizing antibody titers but also significantly enhanced CD8^+^ T-cell responses, indicating unique advantages of heterologous boosting at the cellular immune level (6).

### 3.2 Infection-naive individuals: broad-spectrum immunity through heterologous vaccination strategies

In SARS-CoV-2-naïve individuals, a heterologous prime-boost vaccination strategy can effectively mimic or construct a broad and potent immune response, thereby circumventing the risks associated with natural infection. Research by Awadalla et al. demonstrated that in infection-naive individuals, a heterologous boosting regimen combining mRNA vaccines and viral vector vaccines, compared to a homologous regimen, not only induced higher levels of anti-S protein IgG antibodies but, more importantly, elicited significantly greater ACE2 receptor-binding inhibition against the Omicron B.1.1.529 variant and stimulated a more robust CD8^+^T cell IFN-*γ* response (5). Elliott et al. also incorporated infection-naïve individuals in the aforementioned study for comparative analysis, the heterologous combination of saRNA and mRNA, while yielding slightly lower antibody titers than in convalescent individuals, still induced significant T-cell responses, with the proportion of CD8^+^T cells being notably higher than in the homologous mRNA group (6). This strategy can induce broad-spectrum responses resembling hybrid immunity without the risks of infection, achieving optimization particularly in cellular immunity and tissue-resident memory (26). Consistent with this, a recent prospective study by Tseng et al. further demonstrated that heterologous vaccination regimens not only elicit stronger humoral immunity but also correlate with reduced breakthrough infection rates in healthy adults, providing updated real-world evidence for their application in infection-naïve populations (27).

### 3.3 Breakthrough infections: immune augmentation by heterologous prime-boost regimens

Final results from the PRIBIVAC (Pre-booster Immunogenicity and Booster Impact of Vaccine Against COVID-19) clinical trial illuminate the path to constructing the strongest hybrid immunity: among individuals who completed mRNA vaccine primary series, heterologous boosting (e.g., with mRNA-1273) established a higher baseline immune level compared to homologous boosting; subsequent Omicron breakthrough infection on this elevated baseline not only significantly increased antibody titers but also substantially broadened the breadth of the immune response, inducing potent cross-neutralizing capacity against variants like BA.5 (3). This suggests that the high-level immune baseline established by heterologous boosting, combined with the immune breadth expansion brought by natural infection, collaboratively shapes an optimized hybrid immune state in terms of both breadth and potency.

### 3.4 Clinical effectiveness and safety considerations: evidence from real-world studies

Although heterologous vaccination has demonstrated significant advantages in immunogenicity, its real-world clinical protective efficacy against breakthrough infections and severe disease warrants careful evaluation. A systematic review and meta-analysis encompassing 29 randomized controlled trials with a total of 12,538 adults showed that, compared with homologous booster regimens, heterologous vaccination was not associated with a statistically significant reduction in the risk of laboratory-confirmed symptomatic COVID-19 (RR 0.95, 95% CI 0.72–1.25) or severe COVID-19 (RR 0.51, 95% CI 0.20–1.33), with very low certainty of evidence and insufficient sample size to draw definitive conclusions (28). Regarding safety, heterologous vaccination was associated with an increased risk of non-serious adverse events (RR 1.19, 95% CI 1.08–1.32), whereas the risk of serious adverse events did not differ significantly from that observed with homologous vaccination. A large retrospective cohort study from Gyeonggi Province, South Korea, further indicated that among patients who experienced serious adverse events, heterologous vaccination (viral vector priming followed by mRNA boosting) was associated with an increased 42-day mortality rate (aHR 1.72, 95% CI 1.06–2.78), along with higher incidences of serious adverse events related to respiratory and genitourinary system disorders (29). In addition, animal studies have demonstrated that aluminum-adjuvanted inactivated vaccines can induce vaccine-associated enhanced respiratory disease upon heterologous SARS-CoV-2 challenge, a risk that can be effectively mitigated by adjuvant selection (e.g., RIBI adjuvant) (30). Taken together, although heterologous vaccination offers theoretical advantages in immunogenicity, the evidence supporting its clinical protective efficacy and safety remains insufficient. Future validation through larger, longer-term randomized controlled trials will be essential, particularly in older adults and immunocompromised populations.

## 4 Summary and outlook

The central argument of this review is that the active deployment of a heterologous vaccination strategy not only simulates the advantages of hybrid immunity for infection-naïve individuals, but also optimizes the breadth and durability of immune responses in those who have already acquired hybrid immunity. This strategy aims to transform the uncontrollable risk of natural infection into a designable and implementable immune-boosting regimen, thereby systematically establishing a broader-spectrum, more potent, and longer-lasting population-wide immune barrier. The key mechanism for its success lies in the clever utilization of antigenic distance. When the immune system is sequentially exposed to cognate antigens derived from different technological platforms, subtle differences in conformation, modification, or presentation method constitute a critical immune stimulus (24). These differences help mitigate the constraints of immune imprinting established by the primary immunization, driving the diversification of the B-cell receptor repertoire and affinity maturation, thereby generating neutralizing antibodies with broader cross-reactivity. More importantly, heterologous vaccination uniquely optimizes the CD8 + T-cell response, inducing a higher proportion and more functional effector T cells, which is difficult to achieve with single immunization modalities and constitutes a key mechanism by which hybrid immunity provides durable protection and effectively prevents severe disease. Therefore, systematically integrating the principle of heterologous vaccination into future vaccine development and immunization strategies offers a promising approach for building resilient population immunity. First, future research should move beyond simple antigen updates (e.g., shifting to XBB lineages) and instead focus on exploring the optimal combination sequences of different innovative vaccine platforms (e.g., saRNA, recombinant protein, live-attenuated vaccines) within heterologous strategies. Determining which platform combinations maximize the benefits of antigenic distance is a pressing scientific question. Second, a deeper understanding is warranted of the potential and mechanisms of heterologous vaccination in inducing key protective immune responses such as mucosal immunity and tissue-resident memory. Given the characteristics of respiratory viruses, establishing a powerful first line of defense at the portal of entry is decisive for effectively blocking infection and transmission.

Although this review focuses on SARS-CoV-2, the core principles underlying heterologous vaccination extend beyond COVID-19 and hold potential applicability to other challenging infectious diseases. In the field of tuberculosis, a 2026 study developed a heterologous immunization regimen using recombinant BCG (rBCG) as a priming vector and a recombinant protein (rRBD) as a booster vaccine. This approach not only induced high-titer IgG antibodies but also elicited durable CD4^+^ and CD8^+^ T-cell immune responses, and the addition of an LTB adjuvant resulted in cross-neutralizing activity against the Omicron variant (31). In the malaria field, researchers employed a heterologous prime-boost strategy using a modified vaccinia virus for priming and an adeno-associated virus (AAV1) for boosting to construct a bivalent vaccine targeting both Plasmodium falciparum and Plasmodium vivax. This heterologous immunization regimen provided 70% protective efficacy, achieved 90% transmission-blocking activity, and sustained immune responses for over seven months (32). Collectively, these studies support heterologous vaccination as a versatile strategy to counter the complex immune evasion mechanisms of pathogens. Despite remaining challenges in real-world data accumulation and public health implementation, efforts should shift from passively reacting to viral evolution toward proactively shaping systematic immune defense through the strategic deployment of heterologous vaccination. This approach offers a path to establish population-wide immune protection characterized by breadth, potency, and resilience, enabling a composed and effective response to the endless evolution of the virus.
